# New-Onset Atrial Fibrillation in Patients With Primary Aldosteronism Receiving Different Treatment Strategies: Systematic Review and Pooled Analysis of Three Studies

**DOI:** 10.3389/fendo.2021.646933

**Published:** 2021-05-24

**Authors:** Cheng-Hsuan Tsai, Ya-Li Chen, Chien-Ting Pan, Yen-Tin Lin, Po-Chin Lee, Yu-Wei Chiu, Che-Wei Liao, Zheng-Wei Chen, Chin-Chen Chang, Yi-Yao Chang, Chi-Sheng Hung, Yen-Hung Lin

**Affiliations:** ^1^ Department of Internal Medicine, National Taiwan University Hospital Jin-Shan Branch, New Taipei City, Taiwan; ^2^ Department of Internal Medicine, National Taiwan University Hospital and National Taiwan University College of Medicine, Taipei, Taiwan; ^3^ Department of Internal Medicine, National Taiwan University Hospital Yun-Lin Branch, Yun-Lin, Taiwan; ^4^ Department of Internal Medicine, Taoyuan General Hospital, Taoyuan, Taiwan; ^5^ Department of Medical Imaging, National Taiwan University Hospital and National Taiwan University College of Medicine, Taipei, Taiwan; ^6^ Cardiology Division of Cardiovascular Medical Center, Far Eastern Memorial Hospital, New Taipei City, Taiwan; ^7^ Department of Computer Science and Engineering, Yuan Ze University, Taoyuan City, Taiwan; ^8^ Department of Medicine, National Taiwan University Cancer Center, Taipei, Taiwan

**Keywords:** hyperaldosteronism, primary aldosteronism, adrenalectomy, mineralocorticoid receptor antagonist, atrial fibrillation

## Abstract

**Background:**

Primary aldosteronism (PA) is a common cause of secondary hypertension and associated with higher incidence of new-onset atrial fibrillation (NOAF). However, the effects of surgical or medical therapies on preventing NOAF in PA patents remain unclear. The aim of this meta-analysis study was to assess the risk of NOAF among PA patients receiving mineralocorticoid receptor antagonist (MRA) treatment, PA patients receiving adrenalectomy, and patients with essential hypertension.

**Methods:**

We performed the meta-analysis of the randomized or observational studies that investigated the incidence rate of NOAF in PA patients receiving MRA treatment versus PA patients receiving adrenalectomy from database inception until December 01, 2020 which were identified from PubMed, Embase, and Cochrane Library.

**Results:**

A total of 172 related studies were reviewed, of which three fulfilled the inclusion criteria, including a total of 2,705 PA patients. The results of meta-analysis demonstrated a higher incidence of NOAF among the PA patients receiving MRA treatment compared to the PA patients receiving adrenalectomy (pooled odds ratio [OR]: 2.83, 95% confidence interval [CI]: 1.76–4.57 in the random effects model, *I* ^2^ = 0%). The pooled OR for the PA patients receiving MRA treatment compared to the patients with essential hypertension was 1.91 (95% CI: 1.11–3.28). The pooled OR for the PA patients receiving adrenalectomy compared to the patients with essential hypertension was 0.70 (95% CI: 0.28–1.79).

**Conclusion:**

Compared to the essential hypertension patients and the PA patients receiving adrenalectomy, the patients with PA receiving MRA treatment had a higher risk of NOAF.

**Systematic Review Registration:**

https://www.crd.york.ac.uk/prospero/, identifier CRD42021222022.

## Introduction

Primary aldosteronism (PA) is a state of autonomous aldosterone secretion which is unresponsive to renin regulation, resulting in hypertension and electrolyte imbalance (1). The prevalence of PA has been reported to be 4.3 to 9.5% in all patients with hypertension and 17 to 23% in PA patients with resistant hypertension (2, 3). Compared to essential hypertension, PA is associated with higher risks of cardiovascular, renal, and metabolic complications (4–8). The excess aldosterone in PA will cause atrial structural and electrical remodeling which induce atrial fibrillation genesis. A correlation between PA and atrial fibrillation has been identified in previous studies, although the complicated interplay has yet to be completely elucidated (9). Milliez et al. reported that PA patients had a 12.1-fold higher risk of atrial fibrillation compared to essential hypertension patients (10), and a recent meta-analysis reported the risk of atrial fibrillation was 3.5-fold higher in PA patients compared to essential hypertension patients (11).

Atrial fibrillation is the most prevalent arrhythmia among adults that is associated with cerebro-cardiovascular complications (12, 13). The prevalence of atrial fibrillation has been reported to be 1.1% in adults aged above 35 years in Taiwan (14). The prevalence of atrial fibrillation was even higher in the elderly and in patients with chronic illnesses (15). Since that, the detection of new-onset atrial fibrillation (NOAF) is important to allow for timely risk stratification and interventions to prevent stroke or embolic events in PA patients.

Current guideline suggests that PA can be classified as lateralized PA, including aldosterone-producing adenoma and less commonly unilateral hyperplasia, and idiopathic hyperaldosteronism (16). Adrenalectomy is currently the standard treatment for lateralized PA (2, 16–19). However, there are still some PA patients with lateralized disease do not receive adrenalectomy in real world practice due to unwilling to receive surgery or limited equipment or capacity to receive adrenal vein sampling to confirmed the diagnosis (20). For these patients, mineralocorticoid receptor antagonist (MRA) therapy is the alternative treatment strategy for the lateralized PA and MRA is also the suggested treatment strategy for idiopathic hyperaldosteronism (19, 21). Recently, Pan et al. demonstrated that PA patients receiving adrenalectomy have a lower incidence of NOAF than essential hypertension patients and this finding has not been found in PA patients receiving MRA treatment (22).

The findings of these studies indicate that the over-secretion of aldosterone in PA patients may be correlated with the atrial fibrillation genesis, and that the active treatment of PA can reduce the risk of NOAF. Although there is abundant evidence of the strong correlation between PA and atrial fibrillation, few studies have investigated the effects of different treatment strategies on the prevention of NOAF. Therefore, we designed this study to compare the effects of adrenalectomy and MRA treatment on the development of NOAF in PA patients.

## Materials and Methods

### Search Strategy and Selection Criteria

This meta-analysis was performed according to the Preferred Reporting Items for Systematic Reviews and Meta-Analyses (PRISMA) (23). We searched PubMed, Embase, and Cochrane Library for randomized or observational studies using Mesh terms related to PA (e.g. ‘primary aldosteronism’, ‘hyperaldosteronism’, ‘primary aldosteronism/hyperaldosteronism’) and the following terms: ‘adrenalectomy’, ‘atrial fibrillation, arrhythmia’ from the database inception up to December 01, 2020. The studies were examined independently by the same authors as full-text reports according to the following criteria: (i) inclusion of patients with aldosterone-producing adenoma, idiopathic hyperaldosteronism, and essential hypertension; (ii) studies with both adrenalectomy and MRA treatment of patients with PA; (iii) NOAF included as an outcome treatment variable; (iv) exclusion of patients with a history of atrial fibrillation; and (v) limited to human studies. If there was more than one report from the same study group, we selected the report with the largest sample of patients. Review articles or meta-analyses were not included for analysis, but their citations and references were searched for additional relevant studies. The details of the search algorithm were provided in the supplemental materials.

The identified articles were first evaluated at the title or abstract level after consensus between two independent investigators (CHT and YLC). If the articles were potentially relevant, full articles were further retrieved and evaluated as complete reports according to the selection criteria. Studies including prospective clinical trials and retrospective studies were selected if the setting of the studies was to evaluate the outcomes of PA patients comparing the performance of MRA treatment and adrenalectomy. The exclusion criteria were non-human studies, duplicate reports, studies without relevant outcome data, absence of a control group, studies presenting the outcomes of either MRA treatment or adrenalectomy only, or different settings in the intervention and control arms.

### Data Extraction and Quality Assessment

Two independent reviewers (CHT and YLC) extracted the following data: author, journal, year of publication, location of the study group, design of the study, baseline features of the included patients, length of follow-up, numbers of participants enrolled and data of outcome in the studies and those who reached the desired endpoints of the specific studies. For studies reported in more than one publication, data from the most completed publication were extracted.

The quality of the included studies was evaluated using the Risk Of Bias In Non-randomized Studies of Interventions (ROBINS-I) checklist (24), scoring each study for the following seven domains: “Confounding”, “Selection”, “Classification of intervention”, “Deviation from intervention”, “Missing data”, “Measurement of outcomes” and “Selection of repeated results”. The domains were scored as “No information” (0), “Low” (1—low risk of bias), “Moderate” (2—moderate risk of bias), “Serious” (3—Serious risk of bias), and “Critical” (4—Critical risk of bias). A study was categorized as being of high quality if most of the domains were judged to be at low risk of bias.

### Outcomes of Interest

The primary outcome of interest was the risk of NOAF in PA patients receiving MRA therapy versus adrenalectomy. We also analyzed two secondary outcomes: (1) PA patients receiving MRA therapy versus essential hypertension patients; and (2) PA patients receiving adrenalectomy versus essential hypertension patients.

### Statistical Analysis

Data were extracted with the use of a standardized data form. In order to compare differences on the incidence of events between the two treatment groups, we extracted the number of events (NOAF) and total patients in each treatment arm. The pooled odds ratios (OR) with corresponding 95% confidence intervals (CIs) were calculated using inverse variance (IV) fixed and Laird and Ware random effects models with RevMan 5.3 (The Cochran Collaboration, The Nordic Cochrane Centre, Copenhagen, Denmark). The weights in the fixed effects model were assigned according to inverse within-study variance based on the assumption that all studies were sampled from a population with the same effect size. In contrast, the assigned weights in the random effects model considered both within-study and between-study variance.

Heterogeneity across studies was evaluated using the *I* ^2^ index, which was considered to be low if *I* ^2^ was ≤50%, moderate if >50% to <75%, and high if ≥75% (25). We assessed publication bias by visually inspecting funnel plots and Egger’s regression asymmetry test, using Comprehensive Meta-Analysis Version 3.3.070 (Biostat Inc., Englewood, NJ, USA, 2014).

## Results

### Included Studies

**Figure 1:**
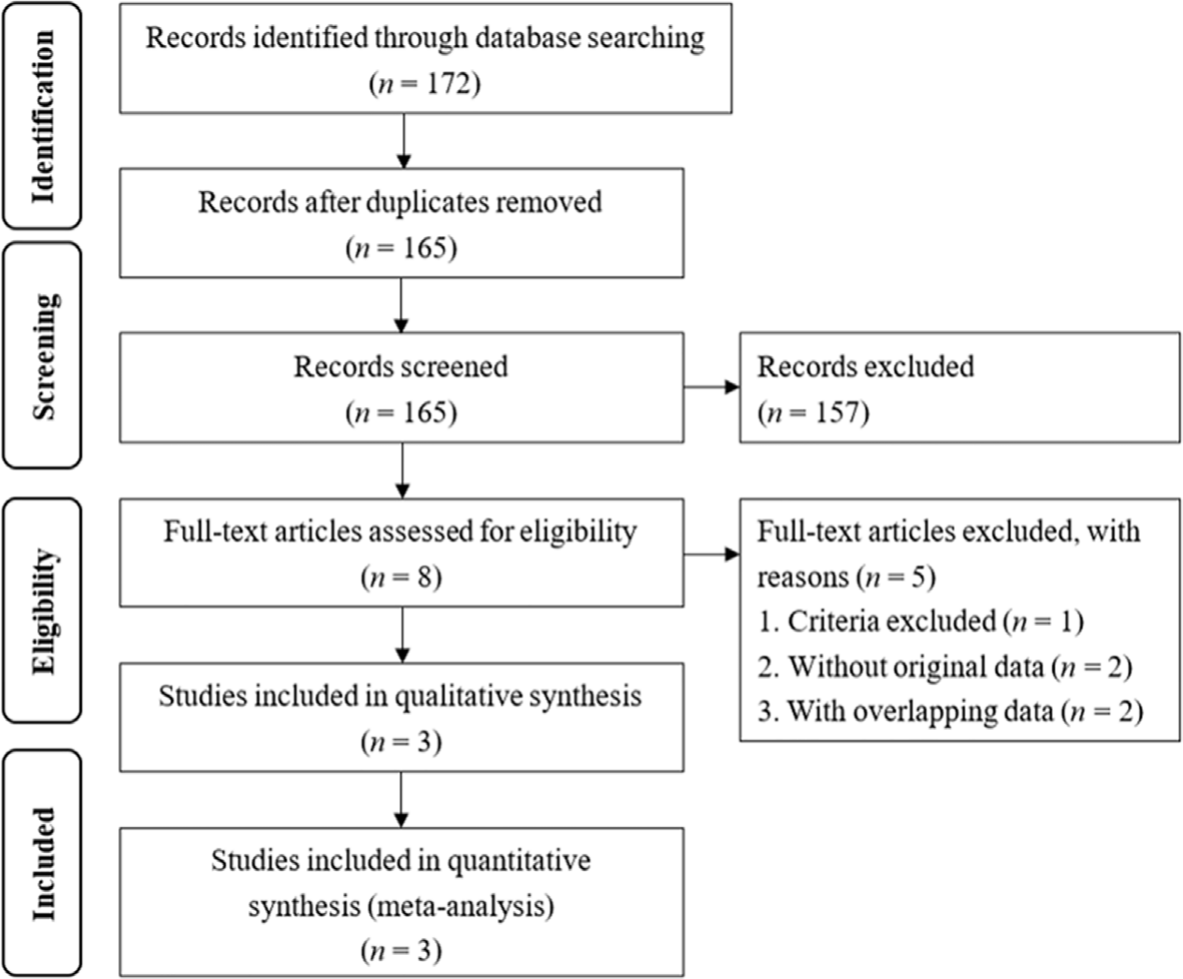
Flow chart of the literature search. The 175 identified studies were from PubMed (11), Embase (152) and Cochrane (9).

The electronic search yielded a total of 172 studies. The references of these studies were examined using the search strategy, and further evaluated for eligibility at the title or abstract level (**Figure 1**). Of the 172 studies assessed, seven were excluded because they were duplicate reports, and 157 studies were excluded after screening the title and abstract. Of the remaining eight full-text articles, five were excluded. In these five excluded studies, one is the editorial comments which not matching the searching criteria and two studies did not have the treatment results of adrenalectomy and MRA treatment. The two studies with overlapping data with current enrolled studies. Compared with current enrolled studies, both excluded studies were the conference abstracts including one with shorter follow-up and another with the same result. One study from our study group did not include details of the number of NOAF events in the study (22). We enrolled the raw data form our study to perform the meta-analysis. Finally, three studies were included in the final meta-analysis (22, 26, 27). In total, these studies included 2,705 PA patients, of who 776 received adrenalectomy and 1,929 received MRA treatment, and 49,794 essential hypertension patients.

Descriptive summaries of each included study are shown in **Table 1**. The types of study included one prospective study (26) and two retrospective studies (22, 27). In these three studies, the median duration of follow-up was 10 years (IQR 7.2–10.9). The dosages of spironolactone used in the MRA treatment groups were listed in **Table 1**.

**Table 1:** Characteristics of the studies meeting the inclusion criteria.

Reference	Study nature	Country	Number of patients			Duration of follow-up* (years)	Initial MRA Dosage	Exclusion criterion
			PA		EH			
			MRA	adrenalectomy				
Hundemer, (27)	Retrospective	USA	195	201	40,092	10	43 to 50 mg^†^	AF, CHF, MI, Stroke
Pan, (12)	Retrospective	Taiwan	1,668	534	8,808	4.4	50 mg	AF, MVD, Hyperthyroidism
Rossi, (26)	Prospective	Italy	66	41	894	11.8	/	AF, secondary hypertension

AF, atrial fibrillation; CHF, congestive heart failure; EH, essential hypertension; MI, myocardial infarction; MRA, mineralocorticoid receptor antagonist; MVD, mitral valve disease; PA, primary aldosteronism.

*For retrospective studies, the duration of follow-up is indicated as mean value; Prospective study is indicated as medium value.

^†^The initial prescription dose of spironolactone was 43 mg in PRA >1 group and 50 mg in PRA <1 group.

### Risk of Bias Assessment

The results of ROBINS-I tool analysis showed that the overall risk of bias of all three included studies were moderate (2) (**Supplement Table**).

### Outcomes

Associations among PA patients receiving MRA treatment, PA patients receiving adrenalectomy, and essential hypertension patients with regards to NOAF events were analyzed.

#### Primary Outcome: Risk of NOAF in the PA Patients Receiving MRA Treatment Compared to the PA Patients Receiving Adrenalectomy

**Figure 2:**
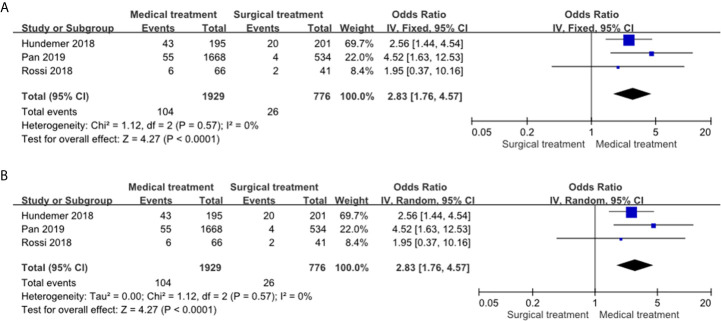
Forest plots of NOAF in PA patients receiving MRA treatment vs adrenalectomy. Forest plots for the fixed effects model **(A)** and random effects model **(B)**. CI, confidence interval; OR, odds ratio; NOAF, new-onset atrial fibrillation; PA, primary aldosteronism; MRA, mineralocorticoid receptor antagonist.

MRA treatment was significantly associated with a higher incidence of NOAF compared to adrenalectomy in both the fixed effects model (OR: 2.83, 95% CI: 1.76–4.57) (**Figure 2A**) and random effects model (OR: 2.83, 95% CI: 1.76–4.57) (**Figure 2B**). The heterogeneity of the included studies was low (I^2^ = 0). The funnel plot (**Supplement Figure**) was generally symmetry and the Egger’s regression asymmetry test (p = 0.91) did not reveal a statistical significance.

#### Risk of NOAF in the PA Patients Receiving MRA Treatment Compared to the Essential Hypertension Patients

**Figure 3:**
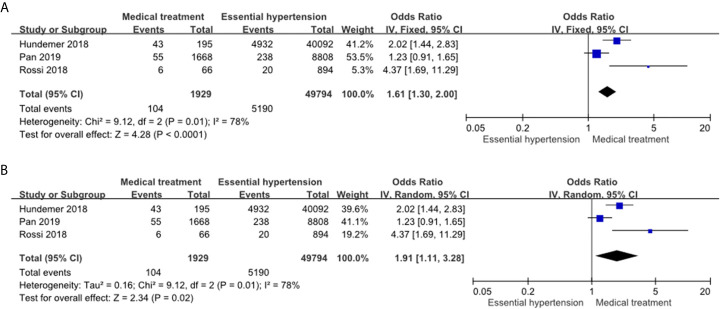
Forest plot of NOAF in PA patients receiving MRA treatment vs EH patients. Forest plots for the fixed effects model **(A)** and random effects model **(B)**. CI, confidence interval; OR, odds ratio; NOAF, new-onset atrial fibrillation; PA, primary aldosteronism; MRA, mineralocorticoid receptor antagonist; EH, essential hypertension.

The PA patients receiving MRA treatment had a significantly higher incidence of NOAF events compared to the essential hypertension patients in the fixed effects model (OR: 1.61, 95% CI: 1.30–2.00) (**Figure 3A**) and random effects model (OR: 1.91, 95% CI: 1.11–3.28) (**Figure 3B**). Of note, the heterogeneity of this comparison was high (*I* ^2^ = 78%).

#### Risk of NOAF in the PA Patients Receiving Adrenalectomy Compared to the Essential Hypertension Patients

**Figure 4:**
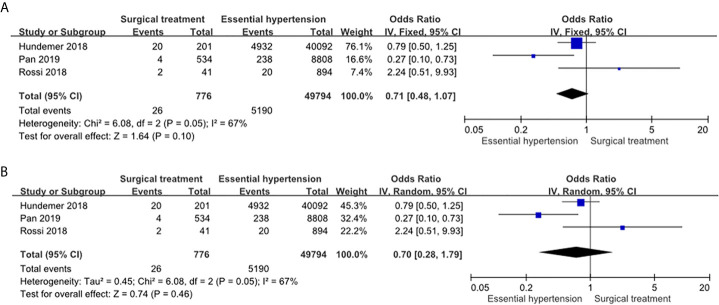
Forest plot of NOAF in PA patients receiving adrenalectomy vs EH patients. Forest plots for the fixed effects model **(A)** and random effects model **(B)**. CI, confidence interval; OR, odds ratio; NOAF, new-onset atrial fibrillation; PA, primary aldosteronism; EH, essential hypertension.

The PA patients receiving adrenalectomy had similar risk of NOAF compared to the essential hypertension patients in the fixed effects model (OR: 0.71, 95% CI: 0.48–1.07) (**Figure 4A**) and in the random effects model (OR: 0.70, 95% CI: 0.28–1.79) (**Figure 4B**). The heterogeneity of this comparison was moderate (*I* ^2^ = 67%).

## Discussion

This is the first meta-analysis to compare the long-term risk of NOAF among PA patients receiving MRA treatment, PA patients receiving adrenalectomy, and essential hypertension patients. The pooled results suggested that the PA patients receiving MRA treatment had a higher risk of NOAF compared to the PA patients receiving adrenalectomy and the patients with essential hypertension. In addition, there was no significant difference in the risk of NOAF between the PA patients receiving adrenalectomy and the essential hypertension patients. These results provide strong evidence of the higher long-term risk of NOAF in PA patients receiving MRA treatment compared to PA patients receiving adrenalectomy and essential hypertension patients.

Atrial fibrillation is associated with increased risks of stroke, heart failure and mortality (28). Excessive aldosterone is a major contributing factor to atrial fibrillation genesis (9, 12), as shown in the German Conn’s Registry which reported a prevalence rate of atrial fibrillation of 7.1% among 553 PA patients (29). Seccia et al. also demonstrated that the PA was highly prevalent in hypertensive patients with unexplained atrial fibrillation in prospective appraisal on the prevalence of primary aldosteronism in hypertensive (PAPPHY) study (30). The MRA also significantly reduced new-onset atrial fibrillation and recurrent atrial fibrillation in general patient population in one recent meta-analysis (31). However, the interactions among hypertension, hyperaldosteronism, and atrial fibrillation genesis in PA patients are complex (9, 12).

In bench studies, excessive aldosterone has been shown to stimulate the development of atrial fibrillation through atrial fibrosis and conduction disturbances (32–34). Atrial fibrosis is arrhythmogenic and the atrial fibrillation also promotes atrial fibrosis (35). The aldosterone increases inflammatory cytokines and increased oxidative stress which result in atrial fibrosis (12). In addition, left ventricular remodeling, which is also strongly correlated to atrial fibrillation. Some clinical studies have shown that aldosterone induces left ventricular hypertrophy and fibrosis which is associated with left ventricular diastolic dysfunction (8, 36–40). The left ventricular diastolic dysfunction has a prominent influence on atrial structure and function which also contributed to the atrial fibrillation genesis (8, 12, 41).

Both lateralized adrenalectomy and medical MRAs treatment can reverse left ventricular remodeling and improve outcomes in PA patients. However, lateralized adrenalectomy can potentially achieve a completely biochemical cure by removing the lesion responsible for hyperaldosteronism (42). Furthermore, the adrenalectomy can achieve the completed therapeutic effects more rapidly compared with medical therapy (43). In PA patients received MRA treatment, it takes longer time to observe a greater reduction in left ventricular wall thickness compare to PA patient receiving adrenalectomy (17). In addition, Rossi et al. compared the long-term outcomes of PA patients after adrenalectomy versus PA patients receiving MRA treatment, and reported a potentially lower left ventricular mass index after adrenalectomy (44). Since that, lateralized adrenalectomy is the standard treatment for PA patients who are suitable to receiving surgery (2, 21). In addition, PA patients received lateralized adrenalectomy had lower risk of new-onset diabetes mellitus (45), better quality of life (46) and less osteoporosis (47) compared with those received medical MRAs treatment. Another benefit of adrenalectomy over MRA treatment is that it can decrease the incidence of atrial fibrillation (26). However, the studies which compared the treatment effects from lateralized adrenalectomy or MRAs treatment to lower NOAF occurrence were still limited.

In the current meta-analysis, we enrolled three large cohort studies which showed consistent results that lateralized adrenalectomy had significantly lower NOAF events compared with medical therapy with low heterogeneity in the analysis. In addition, compared with patients with essential hypertension, only lateralized adrenalectomy could neutralize the risk of NOAC due to excess aldosterone but not MRA treatment. However, the heterogeneity of the results was moderate to high in secondary analysis of essential hypertension and PA patients possibly due to the diversity of patient characteristics and the different dosages of MRA use in these studies. The risk of NOAF in PA patients with MRA compared others receiving adrenalectomy or essential hypertension patients may be contributed form the insufficient MRA dosage and treatment effects (27). Due to limited studies, the subgroup analysis was not applicable. In contrast, the result of primary analysis compared MRA treatment to adrenalectomy revealed a very low heterogeneity.

The optimal dosage of spironolactone has yet to be established, and the current recommendation dosage in guidelines is from a daily dose of 12.5 mg with slow titration to a maximum daily dose of 100 mg (2). Catena et al. evaluated the effect of high-dose spironolactone, and found no significant difference in the occurrence of the combined cardiovascular endpoint of myocardial infarction, stroke, revascularization procedures, and sustained arrhythmias between patients with PA who received adrenalectomy and those who received high-dose MRA treatment (daily spironolactone dose: 121 mg) (HR, 1.26; 95% CI, 0.36–4.44; *P* = 0.71) (43). However, higher incidences of drug adverse effects were found in the high-dose spironolactone treatment group such as gynecomastia. In 2018, Hundemer et al. demonstrated that the level of plasma renin activity (PRA) after spironolactone treatment (<1 or ≥1 ng/ml/h) may be associated with NOAF and worse cardiovascular outcomes, and that the level of PRA after MRA treatment may be a better predictor than MRA dosage to predict clinical outcomes (48). To identify the ideal spironolactone dose in each patient, titrating the dose according to the PRA may be a reasonable approach in those PA patients received medical therapy. The dosages of MRA in the two retrospective studies enrolled in this meta-analysis were relatively low. Pan et al. showed the first prescript spironolactone dosage was only 50 and 75 mg as maximum dosage. Hundemer et al. showed that the initial spironolactone dosages were about 43 to 50 mg and were titrated up to 71 to 84 mg during follow-up. The relatively low spironolactone dosages may contribute to the higher risk of NOAF in PA patients receiving medical MRA treatment.

This study has some limitations. First, the choice of treatment is largely depended on lateralization. When diagnosed as aldosterone-producing adenoma, most patients are treated by adrenalectomy, while those diagnosed as idiopathic hyperaldosteronism are usually treated with MRAs. The different nature between two subtypes may influence the incidence of NOAF. However, data of aldosterone-producing adenoma patients receiving only MRA treatment were limited, and the information of different treatments in aldosterone-producing adenoma were not available in cohorts collected in this study. Further randomized study with different treatment strategies (surgery versus MRA therapy) in aldosterone-producing adenoma patients is needed to solve this issue. Second, many important baseline characteristics such as anti-hypertension medication use, the dosage of MRA, body mass index, waistline, and baseline cardiac function were not available in the enrolled studies which could be the confounding factors and the origin of the heterogeneity. In addition, the follow-up durations were varied between three enrolled studies which might interfere with the results. However, the benefit of adrenalectomy was consistent in this pooled analysis. Third, only limited studies have been conducted to investigate the NOAF in PA in different treatment strategies and no randomized control trial has been conducted to investigate this issue. Fourth, the heterogeneity was moderate to high in secondary analysis of essential hypertension and PA patients possibly due to the diversity of patient characteristics and the different dosages of MRA use in these studies. However, due to limited studies, the subgroup analysis or meta-regression could not be done to explore the source of heterogeneity.

## Conclusions

The PA patients receiving MRA treatment had a higher risk of NOAF compared to the PA patients receiving adrenalectomy and the patients with essential hypertension.

## Data Availability Statement

The original contributions presented in the study are included in the article/**Supplementary Material**. Further inquiries can be directed to the corresponding author.

## Author Contributions

Y-HL conceived and designed the experiments. C-HT, Y-LC, C-TP, Y-TL P-CL, C-WL, Z-WC, C-CC, and Y-YC analyzed the data. C-HT, Y-LC, and Y-HL wrote the paper. C-SH and Y-WC made scientific comments on the manuscript. All authors contributed to the article and approved the submitted version.

## Funding

This research was supported by grants from the Ministry of Science and Technology (MOST 105-2314-B-002-122-MY3, MOST 106-2314-B-002-169-MY3 and MOST 107-2314-B-002 -264 -MY3), National Taiwan University Hospital (NTUH 107-A141, 108-A141, 109-A141, 108-N01, 108-S4382, UN108-37), and the Excellent Translational Medicine Research Projects of National Taiwan University College of Medicine, and National Taiwan University Hospital (109C 101-43).

## Conflict of Interest

The authors declare that the research was conducted in the absence of any commercial or financial relationships that could be construed as a potential conflict of interest.
