# Supplementary material for: New-Onset Atrial Fibrillation in Patients With Primary Aldosteronism Receiving Different Treatment Strategies: Systematic Review and Pooled Analysis of Three Studies
Source: Front Endocrinol (Lausanne). 2021 May 24;12:646933. doi: 10.3389/fendo.2021.646933 (PMC8181760; doi:10.3389/fendo.2021.646933)
Supplement: Supplementary file 1 [file DataSheet_1.docx]

**Search Algorithm:**

**Pubmed Search:**

((("Hyperaldosteronism"[Mesh]) OR "Aldosterone"[Mesh]) AND ((("Atrial Fibrillation"[Mesh])) OR "Arrhythmias, Cardiac"[Mesh])) AND ("Adrenalectomy"[Mesh])

**Embase Search:**

('atrial fibrillation'/exp  OR  'heart arrhythmia'/exp) AND 'adrenalectomy'/exp AND ('hyperaldosteronism'/exp OR 'aldosterone'/exp )

**Cochrance Library Search:**

("Hyperaldosteronism"[Mesh] OR "Aldosterone"[Mesh]) AND "Arrhythmias, Cardiac" [Mesh]

**Supplement Table**

**Range of overall assessment by study and bias domains**

|  | Domain 1: confounding | Domain 2: selection | Domain 3: classification of intervention | Domain 4: deviation from interventions | Domain 5: missing data | Domain 6: measurement of outcomes | Domain 7: selection of reported result | ROBINS-I  overall |
| --- | --- | --- | --- | --- | --- | --- | --- | --- |
| Hundemer, 2018 | 2 | 2 | 1 | 2 | 2 | 2 | 1 | 2  Moderate |
| Pan, 2020 | 2 | 2 | 2 | 2 | 2 | 2 | 1 | 2  Moderate |
| Rossi, 2018 | 2 | 2 | 1 | 2 | 2 | 2 | 1 | 2  Moderate |

*NOTE.* Risk of bias assessment: 0 = No information; 1 = Low; 2 = Moderate; 3 = Serious; 4 = Critical

**Supplement Figure**

**Funnel plot of NOAF in PA patients receiving MRA treatment vs adrenalectomy**


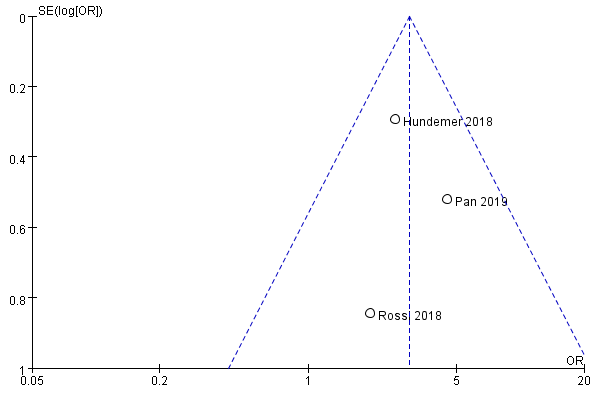


**Abbreviation:** NOAF= new-onset atrial fibrillation; PA=primary aldosteronism; MRA=mineralocorticoid receptor antagonist; OR= odds ratio.
